# Ethyl 5-amino-1-(4-chloro-2-nitro­phen­yl)-1*H*-pyrazole-4-carboxyl­ate

**DOI:** 10.1107/S1600536809000488

**Published:** 2009-01-10

**Authors:** Muhammad Zia-ur-Rehman, Mark R. J. Elsegood, Jamil Anwar Choudary, Muhammad Fasih Ullah, Hamid Latif Siddiqui

**Affiliations:** a Applied Chemistry Research Centre, PCSIR Laboratories Complex, Lahore 54600, Pakistan; b Chemistry Department, Loughborough University, Loughborough, Leicestershire LE11 3TU, England; c Institute of Chemistry, University of the Punjab, Lahore 54590, Pakistan

## Abstract

In the mol­ecule of the title compound, C_12_H_11_ClN_4_O_4_, the pyrazole ring is coplanar with the amino and ethoxy­carbonyl groups within 0.026 (2) and 0.105 (2) Å, respectively. The *C*_6_ ring of the 4-chloro-2-nitro­phenyl group is twisted by 53.58 (4)° relative to the plane of the pyrazole ring. The planar structure of the pyrazole ring is stabilized by an intra­molecular N—H⋯O hydrogen bond between its substituents. Neighbouring mol­ecules are linked through inter­molecular N—H⋯N and N—H⋯O hydrogen bonds, giving rise to one-dimensional tapes along the *b* axis. Mol­ecules in the chain are linked to those of an adjacent chain through weak C—H⋯O inter­actions, forming a three-dimensional network.

## Related literature

For the biological activity of pyrazole and its derivatives, see: Iovu *et al.* (2003 ▶); Mahajan *et al.* (1991 ▶); related literature, see: Akhtar *et al.* (2008 ▶); Baraldi *et al.* (1998 ▶); Bruno *et al.* (1990 ▶); Cottineau *et al.* (2002 ▶); Smith *et al.* (2001 ▶). For the use of pyrazole-based ligands in investigating the structure–activity relationship of the active site of metalloproteins, see: Dardari *et al.* (2006 ▶), and in the preparation of commercially important dyestuffs, see: Baroni & Kovyrzina (1961 ▶); Neunhoeffer *et al.* (1959 ▶). For the synthesis and biological evaluation of heterocyclic compounds, see: Akhtar *et al.* (2008 ▶); Zia-ur-Rehman *et al.* (2006 ▶, 2008 ▶).
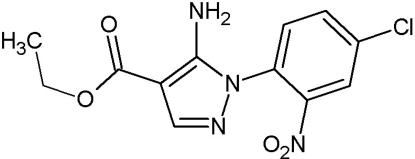


## Experimental

### 

#### Crystal data

C_12_H_11_ClN_4_O_4_
*M* *_r_* = 310.70
Monoclinic, 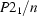

*a* = 8.5899 (8) Å
*b* = 10.2413 (9) Å
*c* = 15.6633 (14) Å
β = 96.5415 (13)°
*V* = 1369.0 (2) Å^3^
*Z* = 4
Mo *K*α radiation
μ = 0.30 mm^−1^
*T* = 150 (2) K
0.79 × 0.27 × 0.09 mm

#### Data collection

Bruker APEXII CCD diffractometer
Absorption correction: multi-scan (*SADABS*; Sheldrick, 2007 ▶) *T*_min_ = 0.797, *T*_max_ = 0.973
15944 measured reflections
4189 independent reflections
3588 reflections with *I* > 2σ(*I*)
*R*_int_ = 0.022

#### Refinement

*R*[*F* ^2^ > 2σ(*F* ^2^)] = 0.034
*wR*(*F* ^2^) = 0.096
*S* = 1.04
4189 reflections
197 parameters
H atoms treated by a mixture of independent and constrained refinement
Δρ_max_ = 0.44 e Å^−3^
Δρ_min_ = −0.27 e Å^−3^

### 

Data collection: *APEX2* (Bruker, 2006 ▶); cell refinement: *SAINT* (Bruker, 2006 ▶); data reduction: *SAINT*; program(s) used to solve structure: *SHELXS97* (Sheldrick, 2008 ▶); program(s) used to refine structure: *SHELXL97* (Sheldrick, 2008 ▶); molecular graphics: *SHELXTL* (Sheldrick, 2008 ▶); software used to prepare material for publication: *SHELXTL* and local programs.

## Supplementary Material

Crystal structure: contains datablocks I, global. DOI: 10.1107/S1600536809000488/bt2841sup1.cif

Structure factors: contains datablocks I. DOI: 10.1107/S1600536809000488/bt2841Isup2.hkl

Additional supplementary materials:  crystallographic information; 3D view; checkCIF report

## Figures and Tables

**Table 1:** Hydrogen-bond geometry (Å, °)

*D*—H⋯*A*	*D*—H	H⋯*A*	*D*⋯*A*	*D*—H⋯*A*
N4—H4*A*⋯O3	0.866 (16)	2.328 (16)	2.9383 (13)	127.7 (12)
N4—H4*A*⋯O2^i^	0.866 (16)	2.610 (15)	3.1356 (13)	120.1 (12)
N4—H4*B*⋯N3^i^	0.871 (15)	2.153 (16)	3.0074 (13)	166.8 (14)

Symmetry code: (i) 
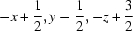
.

**Fig. 1.**
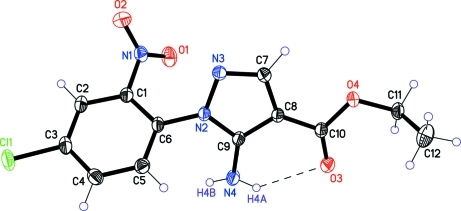
The asymmetric unit of the title compound highlighting the intramolecular H-bond. Displacement ellipsoids are drawn at the 50% probability level.

**Fig. 2.**
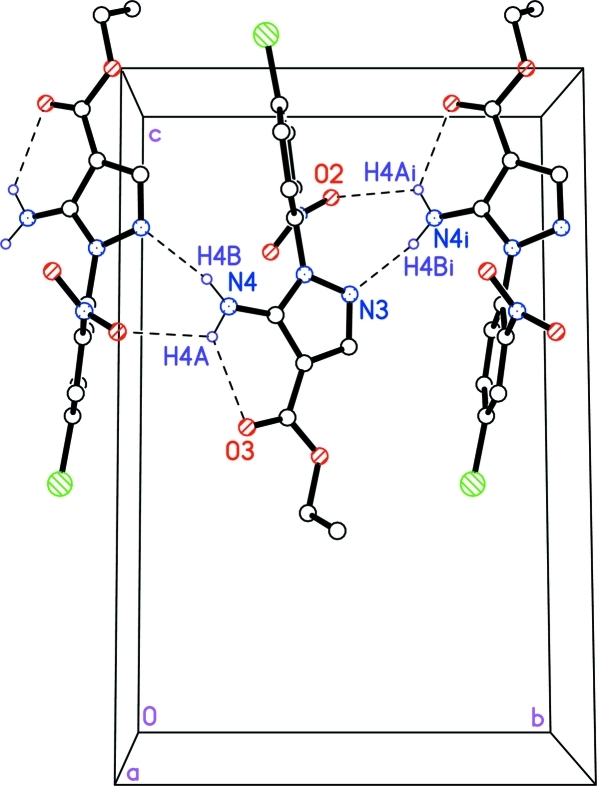
Perspective view of the crystal packing showing hydrogen-bond interactions (dashed lines). H atoms not involved in hydrogen bonding have been omitted for clarity. Symmetry operator i = -x + 1/2, y - 1/2, -z + 3/2.

## supplementary crystallographic information

### Comment

Pyrazole and its derivatives represent one of the most important classes of organic heterocyclic compounds, possessing a wide spectrum of biological activities such as antibacterial, fungicidal (Iovu *et al.*, 2003), herbicidal (Mahajan *et al.*,1991) and antiviral (Baraldi *et al.*, 1998) activities. Some of their derivatives have been reported to possess significant antiarrhythmic & sedative (Bruno *et al.*, 1990), hypoglycemic (Cottineau *et al.*, 2002) and anti-inflammatory (Smith *et al.*, 2001) activities. In addition, pyrazole based ligands have also been used to investigate the structure-activity relationship of the active site of metalloproteins (Dardari *et al.*, 2006) and for the preparation of commercially important dyestuffs (Baroni & Kovyrzina, 1961; Neunhoeffer *et al.*,1959). As part of our ongoing research on the synthesis and biological evaluation of heterocyclic compounds (Akhtar *et al.*, 2008; Zia-ur-Rehman *et al.*, 2006; Zia-ur-Rehman *et al.*, 2008), crystal structure of the title compound, (I) was determined.

In I (Fig. 1) the pyrazole ring is approximately coplanar with the amino and ethyl carboxylate groups. The C_6_ ring of the 4-chloro-2-nitro phenyl group is essentially planar and is twisted by 53.58 (4)° relative to the plane of the pyrazole ring about the C6—N2 bond. The planar structure of the pyrazole ring is stabilized by an intramolecular N—H···O hydrogen bond between the amino and ethyl carboxylate substituents (Table 1). Neighbouring molecules are linked through one N—H···N and one N—H···O intermolecular hydrogen bond giving rise to one-dimensional tapes along the *b* axis (Fig. 2, Table 1). The nitro group is twisted by 37.98 (4)° relative to the C_6_ ring, driven by the desire to form the aforementioned H-bond. Each chain is cross-linked to the next through weak C–H···O interactions.

### Experimental

A mixture of 5-amino-1-(4-chloro-2-nitrophenyl)-1*H*-pyrazole-4-carboxylic acid (3.05 g; 10.0 mmoles), phosphoric acid (0.196 g; 2.0 mmoles) and ethyl alcohol (100 ml) was refluxed for a period of five hours. The reaction mixture was then concentrated (to a volume of 20 ml) by slow distillation of ethanol followed by cooling and addition of cold water. The precipitated solid was then filtered, washed with cold water and dried. Crystals suitable for single-crystal X-ray diffraction were grown by slow evaporation of a solution of the title compound in a mixture of ethanol and water (85: 15); yield: 73.68%.

### Refinement

H atoms bound to C were placed in geometric positions (C—H distance = 0.95 Å) using a riding model. H atoms on N had coordinates freely refined. *U*_iso_ values were set to 1.2*U*_eq_ (1.5*U*_eq_ for CH_3_).

### Crystal data

**Table:**

C_12_H_11_ClN_4_O_4_	*F*(000) = 640
*M**_r_* = 310.70	*D*_x_ = 1.508 Mg m^−^^3^
Monoclinic, *P*2_1_/*n*	Melting point: 435 K
Hall symbol: -P 2yn	Mo *K*α radiation, λ = 0.71073 Å
*a* = 8.5899 (8) Å	Cell parameters from 6502 reflections
*b* = 10.2413 (9) Å	θ = 2.6–30.6°
*c* = 15.6633 (14) Å	µ = 0.30 mm^−^^1^
β = 96.5415 (13)°	*T* = 150 K
*V* = 1369.0 (2) Å^3^	Lath, colourless
*Z* = 4	0.79 × 0.27 × 0.09 mm

### Data collection

**Table:**

Bruker APEXII CCD diffractometer	4189 independent reflections
Radiation source: fine-focus sealed tube	3588 reflections with *I* > 2σ(*I*)
graphite	*R*_int_ = 0.022
ω rotation with narrow frames scans	θ_max_ = 30.6°, θ_min_ = 2.4°
Absorption correction: multi-scan (*SADABS*; Sheldrick, 2007)	*h* = −12→12
*T*_min_ = 0.797, *T*_max_ = 0.973	*k* = −14→14
15944 measured reflections	*l* = −22→22

### Refinement

**Table:**

Refinement on *F*^2^	Primary atom site location: structure-invariant direct methods
Least-squares matrix: full	Secondary atom site location: all non-H atoms found by direct methods
*R*[*F*^2^ > 2σ(*F*^2^)] = 0.034	Hydrogen site location: geom except NH coords freely refined
*wR*(*F*^2^) = 0.096	H atoms treated by a mixture of independent and constrained refinement
*S* = 1.04	*w* = 1/[σ^2^(*F*_o_^2^) + (0.0507*P*)^2^ + 0.3692*P*] where *P* = (*F*_o_^2^ + 2*F*_c_^2^)/3
4189 reflections	(Δ/σ)_max_ = 0.001
197 parameters	Δρ_max_ = 0.44 e Å^−^^3^
0 restraints	Δρ_min_ = −0.27 e Å^−^^3^

### Special details

**Table:**

Geometry. All e.s.d.'s (except the e.s.d. in the dihedral angle between two l.s. planes) are estimated using the full covariance matrix. The cell e.s.d.'s are taken into account individually in the estimation of e.s.d.'s in distances, angles and torsion angles; correlations between e.s.d.'s in cell parameters are only used when they are defined by crystal symmetry. An approximate (isotropic) treatment of cell e.s.d.'s is used for estimating e.s.d.'s involving l.s. planes.
Refinement. Refinement of *F*^2^ against ALL reflections. The weighted *R*-factor *wR* and goodness of fit *S* are based on *F*^2^, conventional *R*-factors *R* are based on *F*, with *F* set to zero for negative *F*^2^. The threshold expression of *F*^2^ > σ(*F*^2^) is used only for calculating *R*-factors(gt) *etc*. and is not relevant to the choice of reflections for refinement. *R*-factors based on *F*^2^ are statistically about twice as large as those based on *F*, and *R*- factors based on ALL data will be even larger.

### Fractional atomic coordinates and isotropic or equivalent isotropic displacement parameters (Å^2^)

**Table:**

	*x*	*y*	*z*	*U*_iso_*/*U*_eq_	
C1	0.16661 (12)	0.37722 (10)	0.87165 (6)	0.01863 (19)	
N1	0.00154 (11)	0.39144 (9)	0.83571 (6)	0.02209 (18)	
O1	−0.04727 (11)	0.32020 (9)	0.77617 (6)	0.0316 (2)	
O2	−0.07682 (10)	0.47313 (9)	0.86871 (6)	0.03110 (19)	
C2	0.19621 (13)	0.36020 (11)	0.95971 (7)	0.0216 (2)	
H2	0.1135	0.3609	0.9951	0.026*	
C3	0.35046 (14)	0.34216 (10)	0.99458 (7)	0.0224 (2)	
Cl1	0.39123 (4)	0.31052 (3)	1.103127 (17)	0.03302 (9)	
C4	0.47238 (13)	0.34677 (11)	0.94341 (7)	0.0229 (2)	
H4	0.5777	0.3370	0.9685	0.028*	
C5	0.43941 (13)	0.36565 (10)	0.85546 (7)	0.0218 (2)	
H5	0.5227	0.3691	0.8205	0.026*	
C6	0.28554 (12)	0.37949 (10)	0.81810 (6)	0.01836 (19)	
N2	0.25381 (11)	0.40263 (9)	0.72882 (5)	0.01940 (17)	
N3	0.16947 (11)	0.51311 (9)	0.69958 (6)	0.02197 (19)	
C7	0.17042 (12)	0.51027 (10)	0.61560 (7)	0.0206 (2)	
H7	0.1215	0.5744	0.5778	0.025*	
C8	0.25215 (12)	0.40156 (10)	0.58811 (6)	0.01772 (19)	
C9	0.30396 (12)	0.33322 (10)	0.66310 (6)	0.01755 (19)	
N4	0.39081 (12)	0.22356 (9)	0.67437 (6)	0.0241 (2)	
H4A	0.4077 (18)	0.1853 (14)	0.6270 (11)	0.029*	
H4B	0.3806 (18)	0.1713 (14)	0.7173 (10)	0.029*	
C10	0.28074 (12)	0.36124 (10)	0.50279 (6)	0.01900 (19)	
O3	0.35936 (10)	0.26670 (8)	0.48774 (5)	0.02577 (17)	
O4	0.20696 (9)	0.43861 (8)	0.44194 (5)	0.02405 (17)	
C11	0.23017 (13)	0.40996 (12)	0.35329 (7)	0.0250 (2)	
H11A	0.1377	0.4396	0.3146	0.030*	
H11B	0.2407	0.3145	0.3460	0.030*	
C12	0.37412 (17)	0.47691 (14)	0.32935 (9)	0.0355 (3)	
H12A	0.3655	0.5711	0.3389	0.053*	
H12B	0.3845	0.4605	0.2686	0.053*	
H12C	0.4665	0.4429	0.3649	0.053*	

### Atomic displacement parameters (Å^2^)

**Table:**

	*U*^11^	*U*^22^	*U*^33^	*U*^12^	*U*^13^	*U*^23^
C1	0.0220 (5)	0.0179 (4)	0.0160 (4)	0.0002 (4)	0.0023 (4)	−0.0002 (3)
N1	0.0232 (4)	0.0248 (4)	0.0183 (4)	−0.0023 (3)	0.0024 (3)	0.0032 (3)
O1	0.0339 (5)	0.0367 (5)	0.0230 (4)	−0.0084 (4)	−0.0021 (3)	−0.0042 (3)
O2	0.0271 (4)	0.0339 (5)	0.0330 (5)	0.0062 (3)	0.0063 (3)	−0.0003 (4)
C2	0.0273 (5)	0.0227 (5)	0.0153 (4)	−0.0007 (4)	0.0047 (4)	−0.0004 (4)
C3	0.0319 (5)	0.0202 (5)	0.0146 (4)	0.0005 (4)	0.0007 (4)	−0.0002 (4)
Cl1	0.04426 (18)	0.03893 (17)	0.01462 (13)	0.00482 (13)	−0.00204 (11)	0.00275 (10)
C4	0.0253 (5)	0.0213 (5)	0.0213 (5)	0.0024 (4)	−0.0011 (4)	0.0009 (4)
C5	0.0243 (5)	0.0208 (5)	0.0209 (5)	0.0029 (4)	0.0054 (4)	0.0020 (4)
C6	0.0256 (5)	0.0164 (4)	0.0134 (4)	0.0016 (4)	0.0035 (4)	0.0008 (3)
N2	0.0258 (4)	0.0188 (4)	0.0142 (4)	0.0057 (3)	0.0046 (3)	0.0019 (3)
N3	0.0291 (4)	0.0192 (4)	0.0179 (4)	0.0085 (3)	0.0044 (3)	0.0019 (3)
C7	0.0237 (5)	0.0207 (5)	0.0174 (4)	0.0043 (4)	0.0030 (4)	0.0022 (4)
C8	0.0202 (4)	0.0185 (4)	0.0149 (4)	0.0013 (3)	0.0038 (3)	0.0015 (3)
C9	0.0205 (4)	0.0171 (4)	0.0156 (4)	0.0007 (3)	0.0048 (3)	0.0004 (3)
N4	0.0361 (5)	0.0188 (4)	0.0187 (4)	0.0091 (4)	0.0090 (4)	0.0037 (3)
C10	0.0193 (4)	0.0223 (5)	0.0157 (4)	−0.0012 (4)	0.0028 (3)	0.0010 (4)
O3	0.0318 (4)	0.0266 (4)	0.0196 (4)	0.0076 (3)	0.0058 (3)	−0.0015 (3)
O4	0.0266 (4)	0.0313 (4)	0.0145 (3)	0.0064 (3)	0.0031 (3)	0.0029 (3)
C11	0.0238 (5)	0.0369 (6)	0.0141 (4)	−0.0005 (4)	0.0019 (4)	0.0012 (4)
C12	0.0395 (7)	0.0374 (7)	0.0321 (6)	−0.0095 (6)	0.0150 (5)	0.0001 (5)

### Geometric parameters (Å, °)

**Table:**

C1—C2	1.3848 (14)	C7—C8	1.4096 (14)
C1—C6	1.3940 (14)	C7—H7	0.9500
C1—N1	1.4719 (14)	C8—C9	1.3958 (14)
N1—O1	1.2202 (13)	C8—C10	1.4462 (14)
N1—O2	1.2245 (13)	C9—N4	1.3487 (13)
C2—C3	1.3868 (16)	N4—H4A	0.866 (16)
C2—H2	0.9500	N4—H4B	0.871 (15)
C3—C4	1.3902 (16)	C10—O3	1.2188 (13)
C3—Cl1	1.7271 (11)	C10—O4	1.3418 (12)
C4—C5	1.3881 (15)	O4—C11	1.4549 (13)
C4—H4	0.9500	C11—C12	1.4985 (17)
C5—C6	1.3904 (15)	C11—H11A	0.9900
C5—H5	0.9500	C11—H11B	0.9900
C6—N2	1.4140 (12)	C12—H12A	0.9800
N2—C9	1.3607 (13)	C12—H12B	0.9800
N2—N3	1.3925 (12)	C12—H12C	0.9800
N3—C7	1.3166 (13)		
			
C2—C1—C6	122.51 (10)	C8—C7—H7	123.8
C2—C1—N1	116.93 (9)	C9—C8—C7	105.13 (9)
C6—C1—N1	120.55 (9)	C9—C8—C10	124.27 (9)
O1—N1—O2	125.00 (10)	C7—C8—C10	130.60 (9)
O1—N1—C1	117.73 (9)	N4—C9—N2	123.64 (9)
O2—N1—C1	117.26 (9)	N4—C9—C8	130.26 (9)
C1—C2—C3	117.89 (10)	N2—C9—C8	106.06 (9)
C1—C2—H2	121.1	C9—N4—H4A	114.1 (10)
C3—C2—H2	121.1	C9—N4—H4B	120.7 (10)
C2—C3—C4	121.15 (10)	H4A—N4—H4B	115.1 (14)
C2—C3—Cl1	119.35 (9)	O3—C10—O4	123.99 (9)
C4—C3—Cl1	119.49 (9)	O3—C10—C8	124.18 (9)
C5—C4—C3	119.67 (10)	O4—C10—C8	111.82 (9)
C5—C4—H4	120.2	C10—O4—C11	116.99 (9)
C3—C4—H4	120.2	O4—C11—C12	110.69 (10)
C4—C5—C6	120.57 (10)	O4—C11—H11A	109.5
C4—C5—H5	119.7	C12—C11—H11A	109.5
C6—C5—H5	119.7	O4—C11—H11B	109.5
C5—C6—C1	118.15 (9)	C12—C11—H11B	109.5
C5—C6—N2	120.01 (9)	H11A—C11—H11B	108.1
C1—C6—N2	121.74 (9)	C11—C12—H12A	109.5
C9—N2—N3	111.92 (8)	C11—C12—H12B	109.5
C9—N2—C6	128.23 (9)	H12A—C12—H12B	109.5
N3—N2—C6	119.72 (8)	C11—C12—H12C	109.5
C7—N3—N2	104.38 (8)	H12A—C12—H12C	109.5
N3—C7—C8	112.50 (9)	H12B—C12—H12C	109.5
N3—C7—H7	123.8		
			
C2—C1—N1—O1	−127.78 (11)	C9—N2—N3—C7	−0.53 (12)
C6—C1—N1—O1	51.47 (14)	C6—N2—N3—C7	175.66 (9)
C2—C1—N1—O2	51.39 (13)	N2—N3—C7—C8	0.14 (12)
C6—C1—N1—O2	−129.37 (11)	N3—C7—C8—C9	0.27 (12)
C6—C1—C2—C3	−1.37 (16)	N3—C7—C8—C10	179.76 (10)
N1—C1—C2—C3	177.85 (9)	N3—N2—C9—N4	178.62 (10)
C1—C2—C3—C4	2.81 (16)	C6—N2—C9—N4	2.83 (17)
C1—C2—C3—Cl1	−176.02 (8)	N3—N2—C9—C8	0.70 (12)
C2—C3—C4—C5	−2.04 (17)	C6—N2—C9—C8	−175.09 (10)
Cl1—C3—C4—C5	176.78 (8)	C7—C8—C9—N4	−178.30 (11)
C3—C4—C5—C6	−0.24 (16)	C10—C8—C9—N4	2.17 (18)
C4—C5—C6—C1	1.62 (16)	C7—C8—C9—N2	−0.57 (11)
C4—C5—C6—N2	178.08 (10)	C10—C8—C9—N2	179.90 (10)
C2—C1—C6—C5	−0.81 (16)	C9—C8—C10—O3	−3.61 (17)
N1—C1—C6—C5	179.99 (9)	C7—C8—C10—O3	176.99 (11)
C2—C1—C6—N2	−177.21 (10)	C9—C8—C10—O4	174.82 (10)
N1—C1—C6—N2	3.59 (15)	C7—C8—C10—O4	−4.59 (16)
C5—C6—N2—C9	52.72 (15)	O3—C10—O4—C11	−3.17 (15)
C1—C6—N2—C9	−130.95 (11)	C8—C10—O4—C11	178.40 (9)
C5—C6—N2—N3	−122.79 (11)	C10—O4—C11—C12	−86.64 (13)
C1—C6—N2—N3	53.54 (14)		

### Hydrogen-bond geometry (Å, °)

**Table:**

*D*—H···*A*	*D*—H	H···*A*	*D*···*A*	*D*—H···*A*
N4—H4A···O3	0.866 (16)	2.328 (16)	2.9383 (13)	127.7 (12)
N4—H4A···O2^i^	0.866 (16)	2.610 (15)	3.1356 (13)	120.1 (12)
N4—H4B···N3^i^	0.871 (15)	2.153 (16)	3.0074 (13)	166.8 (14)

Symmetry codes: (i) −*x*+1/2, *y*−1/2, −*z*+3/2.
